# Papillary muscle avulsion following balloon mitral valvotomy evaluated with three-dimensional echocardiography

**DOI:** 10.1186/s12893-022-01636-6

**Published:** 2022-05-21

**Authors:** Wenjuan Bai, Ling Deng, Hong Tang

**Affiliations:** Department of Cardiology, West China Hospital, Sichuan University, No. 37, Guoxue Street, Wuhou District, Chengdu, 610041 China

**Keywords:** Alvusion, Papillary muscle, Balloon mitral valvotomy

## Abstract

**Background:**

Percutaneous balloon mitral valvotomy is a common therapeutic approach for rheumatic mitral stenosis. Avulsion of the papillary muscle is a rare but serious complication of balloon mitral valvotomy. The papillary muscles are derived from the trabecular layer of the developing ventricular walls. When subjected to a force, avulsion of papillary muscle from the trabecular layer may occur.

**Case presentation:**

In this case report, we describe a patient with rheumatic mitral stenosis, who experienced avulsion of the mitral papillary muscle from the left ventricular wall after undergoing balloon mitral valvotomy. Papillary muscle alvusion resulted in severe mitral regurgitation, which was finally treated by mitral valve replacement.

**Conclusion:**

We successfully diagnosed avulsion of the papillary muscle following balloon mitral valvotomy. Three-dimensional transthoracic echocardiography provides more information on mitral apparatus structure than two-dimensional transthoracic echocardiography.

**Supplementary Information:**

The online version contains supplementary material available at 10.1186/s12893-022-01636-6.

## Background

Percutaneous balloon mitral valvotomy (BMV) is a common therapeutic approach for rheumatic mitral stenosis (MS) [1]. BMV results in significant changes in mitral valve morphology and improves leaflet mobility. However, mitral regurgitation (MR) is still a major concern after BMV. In most patients, MR is usually mild and well tolerated. Severe MR is rare, and its causes include leaflet tearing, rupture of the chorae tendinae or the papillary muscle, and papillary muscle avulsion [2].

## Case presentation

**Fig. 1:**
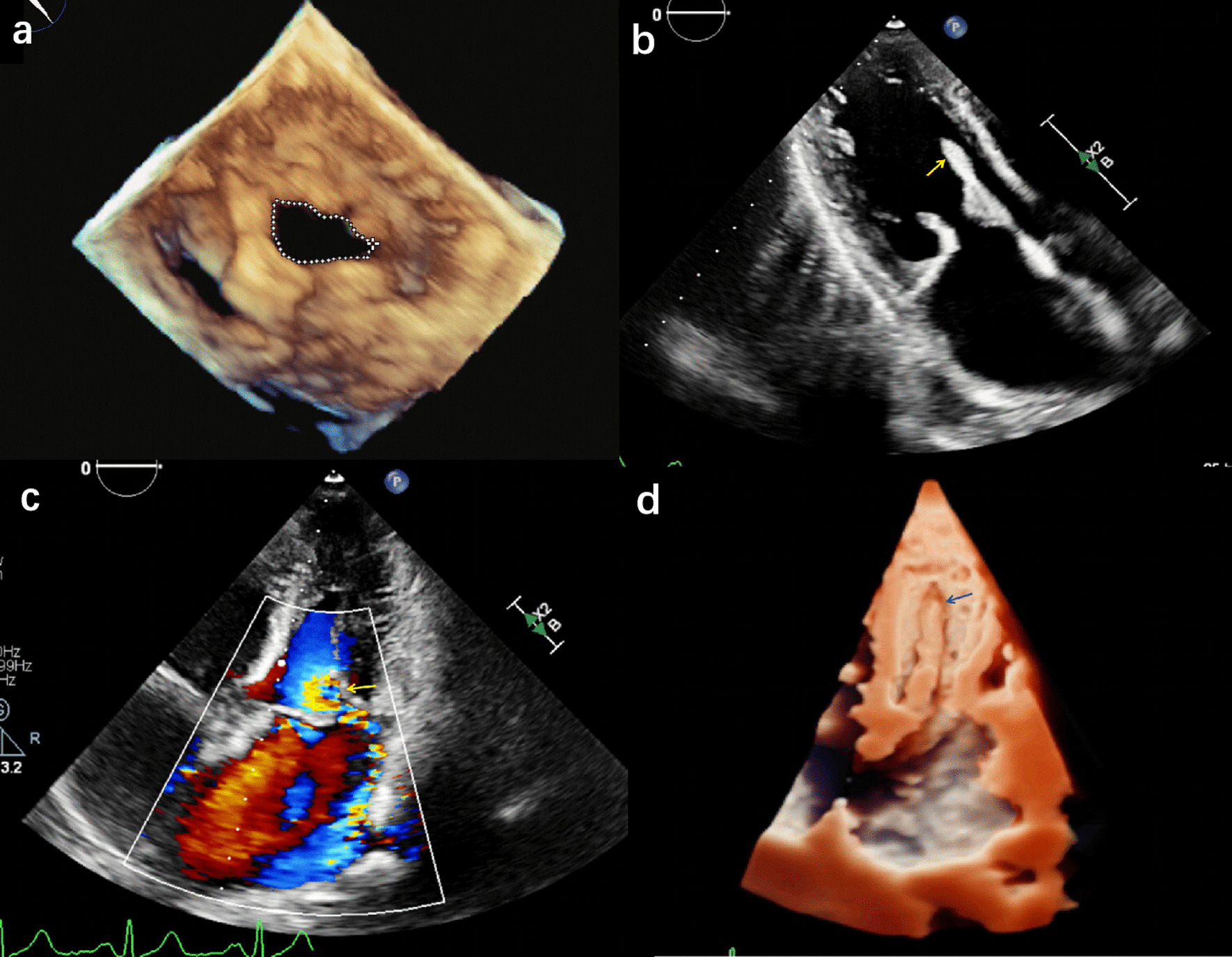
**a** Three-dimensional transesophageal echocardiography revealing mitral stenosis. **b** Transthoracic echocardiography revealing avulsion of the papillary muscle from the left ventricular wall. **c** Color Doppler Flow Imaging revealing severe mitral regurgitation (yellow arrow). **d** Three-dimensional echocardiogram revealing papillary muscle avulsion (blue arrows)

A 49-year-old man presented with exertional dyspnea. Physical examination revealed a loud S1 and a diastolic murmur in the apical region. Transthoracic echocardiography revealed severe rheumatic MS with mild MR, as well as mild functional tricuspid regurgitation and a pulmonary pressure of 55 mmHg. Then, the patient underwent a three-dimensional transesophageal echocardiography to assess the mitral apparatus more accurately and exclude the presence of left atrial appendage thrombus (Fig. 1a). His Wilkins score was 8. This is a borderline score to take BMV. However, the patient prefers to take the percutaneous intervention rather than thoracotomy. Thus, BMV was performed via the antegrade approach under local anesthesia and X-ray fluoroscopy guidance. After transseptal puncture, a 24-mm balloon was used to achieve a single dilatation of 22 mm, after which the mean transmitral pressure gradient decreased from 19 to 11 mmHg. Intraoperative transthoracic echocardiography performed immediately afterwards revealed a significant increase in mitral regurgitation. However, the subvalvular structure could not be observed clearly because the patient was in a supine position. His vital signs remained stable with no definite symptoms, and he refused mitral valve replacement. He was discharged 4 days later after undergoing BMV. Two-dimensional transthoracic echocardiography follow-up performed one month later revealed severe MR caused by a freely mobile part of the mitral papillary muscle (Fig. 1b, c; Additional file 2: Video I). The left ventricular cavity and the two groups of papillary muscle were then imaged using three-dimensional transthoracic echocardiography, which showed avulsion of the posterior papillary muscle (PMA) from the left ventricular trabecular muscle. No residual papillary muscle attachment to the left ventricular wall was observed, confirming avulsion of the posterior papillary rather than its rupture (Fig. 1d; Additional file 3: Video II). In addition, the left ventricular end-diastolic dimension increased from 45 to 53 mm. The estimated systolic pulmonary pressure increased from 55 mmHg to 81 mmHg. Therefore, the patient underwent mechanical mitral valve replacement and tricuspid annuloplasty. After surgery, the patient recovered without symptoms. At 1-month and three-month follow ups, the patient was asymptomatic, and transthoracic echocardiography showed that the left ventricular volume and pulmonary systolic pressure were significantly lower than before surgery.

## Discussion and conclusions

BMV is the preferred treatment for severe MS because it is less invasive approach and low risk. Usually, Wilkins echocardiographic score is used to determine the suitability of the mitral valve structure for BMV [3]. A score of ≤ 8 is considered ideal for BMV. When the score is higher, the chance of significant MR following BMV increases. BMV-related mitral regurgitation may result from leaflet tearing, rupture of the chorae tendinae or the papillary muscle, or PMA, which is an extremely rare complication of BMV. The papillary muscles are derived from the trabecular layer of the developing ventricular walls [4]. When subjected to a force, avulsion of papillary muscle from the trabecular layer may occur. PMA can easily be misdiagnosed as a papillary muscle rupture, which is usually observed as two short residual parts of the muscle attached to the chordae tendineae and the left ventricle separately [5].

Although current strategies for the treatment of PMA and papillary muscle rupture remain the same, many new treatments are being explored, such as artificial chordae tendineae implantation for the treatment of rupture of mitral chordae tendineae [6]. In the future, it is possible that the treatment strategies for these two complications will diverge. Therefore, a clear image of its anatomy is important for selecting the most appropriate therapeutic modality. In our case, three-dimensional echocardiography enhanced the visualization of anatomical details and allowed better delineation of the mitral papillary muscles and the left ventricular endocardium, thereby increasing diagnostic accuracy, although it did not affect treatment choice.

## Supplementary Information

**Additional file 1:** All echocardiography data of the patient.

**Additional file 2:** Video I Two-dimensional echocardiogram revealing the papillary muscle moves extensively in the left ventricle.

**Additional file 3:** Video II Dual-volume 3-dimensional volume-rendering echocardiogram showing two half-hearts simultaneously. No attachment of the residual papillary muscle to the ventricular wall is visible.

## Data Availability

All data generated or analyzed during this study was uploaded as Additional file 1. The datasets used during this study are available from the corresponding author on reasonable request.

## Abbreviations

BMV: Balloon mitral valvotomy
MS: Mitral stenosis
MR: Mitral regurgitation
PMA: Papillary muscle avulsion
